# Burden of tuberculosis in Eastern Africa region from 1990–2021: A systematic analysis for the Global Burden of Disease 2021 Study

**DOI:** 10.1371/journal.pone.0331035

**Published:** 2025-09-02

**Authors:** Leltework Yismaw, Temesgen Zewotir, Essey Kebede Muluneh, Fentabil Getnet, Kerebih Getinet, Habtamu Abebe Getahun, Taye Abuhay Zewale, Mengistie Kassahun Tariku, Anemaw Asrat, Mulusew Andualem, Awoke Misganaw

**Affiliations:** 1 Department of Public Health, College of Health Science, Debre Markos University, Debre Markos, Ethiopia; 2 Department of Epidemiology and Biostatistics, School of Public Health, Bahir Dar University, Bahir Dar, Ethiopia; 3 School of Mathematics, Statistics and Computer Science, University of KwaZulu-Natal, Durban, South Africa; 4 National Data Management Center for Health, Ethiopian Public Health Institute, Addis Ababa, Ethiopia; 5 Department of Global Health and Population, Harvard T.H. Chan School of Public Health, Boston, Massachusetts, United States of America; 6 Department of Computer Science, Debre Markos University, Debre Markos, Ethiopia; 7 Department of Computer Science, Faculty of Computing, Institute of Technology, Bahir Dar University, Bahir Dar, Ethiopia; 8 Department of Epidemiology and Biostatistics, Institute of Public Health, College of Medicine and Health Science, University of Gondar, Gondar, Ethiopia; 9 Department of Health System and Project Management, Collage of Medicine and Health Science, Bahir Dar University, Bahir Dar, Ethiopia; 10 Ethiopian Public Health Institute, Addis Ababa, Ethiopia; 11 Department of Health Metrics Sciences, University of Washington, Seattle, Washington, United States of America; Debre Tabor University, ETHIOPIA

## Abstract

**Background:**

Tuberculosis (TB), despite being a preventable and curable disease, remains a leading infectious cause of death. In Eastern Africa, TB poses a significant public health challenge. This study examined TB incidence, prevalence, mortality, and disability-adjusted life years (DALYs) from 1990 to 2021. This study aims to provide evidence for policy and healthcare stakeholders in Eastern Africa.

**Method:**

This analysis is part of the Global Burden of Disease (GBD) Study 2021 to estimate TB incidence, prevalence, TB-specific mortality, and DALYs. The GBD study applies several analytical tools and uses data from national health surveys, vital registration systems, WHO reports, and hospital records. The results were presented by age group, sex, location, and year, accounting for 95% uncertainty intervals.

**Result:**

A significant decline was observed in TB burden across East African countries between 1990 and 2021. The age standardized TB incidence rate dropped by 53% (95% UI: 50.7%, 55.1%), from 518.8 per 100,000 in 1990–244 in 2021, while TB prevalence dropped by 29.1% (95% UI: 26.3%, 31.7%), from 38,577.6–27,366.1 per 100,000. TB-related deaths fell by 64.6% (95% UI: 55.0%, 71.4%), and TB related DALYs declined by 68.2% (95% UI: 60.3%, 73.6%). Despite these improvements, men consistently experienced higher TB incidence, prevalence, mortality, and DALYs compared to women. Ethiopia showed the highest reductions in terms of TB-related mortality and DALYs compared to countries in the region, with annual reduction rates of 6.0% and 6.6%, respectively. Conversely, Somalia had the highest TB burden in 2021 in terms of incidence, mortality, and DALYs. Mauritius and Seychelles maintained the lowest TB burden, attributed to strong health systems and socio-economic conditions.

**Conclusion:**

A significant decline was observed in TB burden across eastern African countries between 1990 and 2021. However, TB rates remain significantly higher than global and African averages. Therefore, continued investment in health systems and tailored interventions is essential to alleviate the disease burdens, specifically in high-prevalence areas.

## Introduction

Tuberculosis (TB), caused by the *Mycobacterium tuberculosis* bacillus, spreads through the air when an infected person coughs or sneezes. The disease primarily affects the lungs (pulmonary TB), but it can affect other body parts as well [1–4]. Despite being a preventable and curable disease, it remains the world’s leading cause of death from a single infectious agent. More than 10 million people fall ill with TB every year [1,5–8]. The World Health Organization (WHO) and nations across the world have agreed to take the necessary actions to end TB by 2030 [9–13]. The Sustainable Development Goals (SDG target 3) set an ambitious goal of ending the TB epidemic by 2030, with interim benchmarks such as a 10% annual reduction in TB incidence worldwide [14–16]. Achieving this goal requires thorough assessment, tracking, and evaluation of progress towards this goal to inform effective policies and programmatic actions [16–18].

Despite these global targets, the TB burden remains a major public health concern in Africa, which contributes a significant share of the global TB burden [19,20]. Several eastern African countries are among the TB high-burdened countries globally. The eastern African countries consistently report high TB incidence rates, including Ethiopia, Kenya, Tanzania, Uganda, and Somalia [21,22]. High rates of HIV co-infection, poverty, weak healthcare systems, drug resistance, and limited access to treatment are some of the factors contributing to the substantial TB burden in Africa [1,19,23,24].

While existing studies provide valuable insights into TB burden and trends at the global, continental, and national levels, there is a critical need for region-specific analyses to better understand the evolving dynamics of TB trends and disparities across countries in the eastern Africa region. This study addresses this gap by providing a systematic analysis of TB burden and trends in the region to support policymakers and healthcare-implementing stakeholders in designing targeted and effective TB control programs [25,26].

## Methods

### Overview

**Fig 1:**
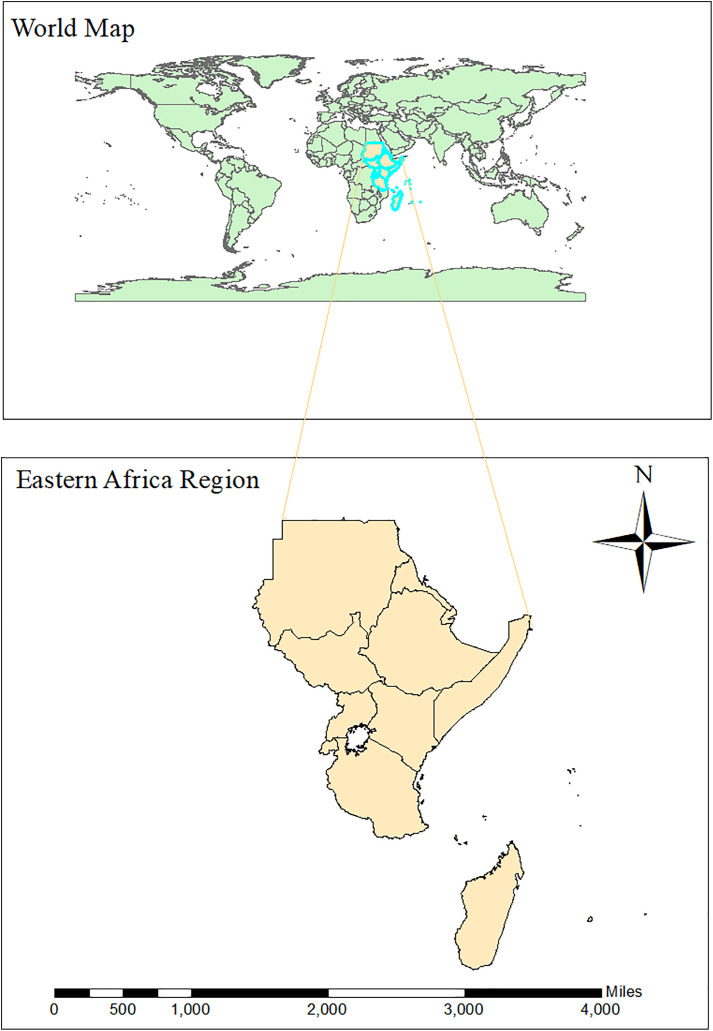
Map of Eastern Africa region based on GBD classification. Base map data from Natural Earth (public domain, https://www.naturalearthdata.com).

In the GBD study, Eastern Africa consists of 14 countries: Ethiopia, Kenya, Uganda, Tanzania, Rwanda, Somalia, South Sudan, Sudan, Djibouti, Eritrea, Comoros, Madagascar, Mauritius, and Seychelles (Fig 1). The region is facing one of the fastest-growing populations globally, with over 450 million inhabitants, a very high fertility rate, and a youth-dominated population structure [27]. Socioeconomic conditions vary widely, with some countries experiencing economic growth while others struggle with poverty, political instability, and conflict [28]. In Eastern Africa, healthcare access is compromised mostly by infrastructure deficiencies, shortages of trained and skilled health workforce, and financial constraints [29]. Primary health care (PHC) systems, being the backbone of health service delivery, are beset by inconsistent coverage, particularly in remote and hard-to-reach areas. Universal health coverage (UHC) policies have been adopted by most countries within the region, but implementation remains problematic due to lack of funds, poor health system governance, and reliance on out-of-pocket payments [30]. Despite these challenges, global initiatives on the fight against TB, such as the WHO End-TB Strategy, the national TB control programs, and increased access to diagnostic tools and treatment, have reinforced the fight against TB [31].

This paper is part of the GBD 2021 collaborative study. The GBD 2021 methodology, extensively detailed elsewhere [32], provides a comprehensive analysis of TB burden across 204 countries, including 14 eastern African countries, from 1990 to 2021. The GBD study applies the Cause of Death Ensemble model (CODEm), which integrates multiple statistical models to accurately estimate cause-specific mortality rates. For TB mortality and years of life lost (YLL), trends were analyzed by age, sex, location, and year, and statistical uncertainty was taken into account with the 1000-draw distribution approach. The inclusion of TB-specific results augmentation and mortality modelling enhances the accuracy of disease burden estimates [33–35]. This describes the techniques that we employed to analyze the tuberculosis burden for GBD 2021.

The GBD study uses a hierarchical classification system to systematically organize a wide spectrum of health conditions based on their fatal and non-fatal impacts. TB is classified under the Communicable, Maternal, Neonatal, and Nutritional (CMNN) diseases category, and is further categorized as a major infectious disease at Level 3 [33].

### Case definition

TB is defined as an infectious disease caused by *the Mycobacterium tuberculosis* complex. It includes all forms of TB, both pulmonary and extra-pulmonary, bacteriologically confirmed or clinically diagnosed. The case definition follows the International Classification of Diseases (ICD)-10 codes and includes A10–19.9, B90–90.9, K67.3, K93.0, M49.0, and P37.0, and the ICD-9 codes are 010–19.9, 137–37.9, 138.0–38.9, and 730.4–30.6. For HIV–tuberculosis, the ICD 10 code is B20.0 [36].

### Data sources

The GBD Study integrates a variety of data sources to generate TB-related mortality estimates using comprehensive approaches. These data sources include national health surveys, vital registration systems, verbal autopsy records, hospital records, WHO reports on TB, and scientific publications [37]. Therefore, YLDs are estimated across all GBD-defined locations and years, enabling temporal and spatial trends to be analyzed. This approach helps identify geographic disparities and track progress toward TB control goals. YLD analysis provides a more complete understanding of the overall disease burden caused by TB by complementing mortality estimates.

### Ethical approval

This is an observational database analysis that uses the Global Burden of Diseases, Injuries, and Risk Factors Study 2021 as the source of information. There was no requirement for ethics approval and consent to conduct this study.

### Mortality estimation method

GBD estimates TB-related mortality using advanced models and analytical approaches. The Cause of Death Ensemble Modelling (CODEm) framework was employed to generate TB mortality estimates, which include key factors such as access to healthcare, adult HIV percentage, smoking rates, and high rank estimates on malnutrition [38]. To improve accuracy, the GBD study incorporates expert input and capture-recapture techniques to address underreporting and misclassification of TB deaths, making sure that TB-related deaths misclassified to other causes, such as pneumonia, are properly corrected [39]. These factors are quantified using population attributable fractions derived from exposure levels and relative risks. The estimates are presented with 95% uncertainty intervals (UIs), accounting for variability in data sources, model parameters, and covariates, ensuring TB mortality estimates are comparable across locations and years [37].

### Incidence and prevalence estimation

Incidence refers to the number of new TB cases in a specific period, while prevalence represents the total number of TB cases at a given time, which is incidence plus duration of illness. To estimate TB incidence, a Bayesian multiphase meta-regression (DisMod-MR) model is used, which simulates state transitions of the disease, including infection, progression, remission, and mortality. Both incidence and prevalence are reported with 95% UIs. These metrics paint a complete picture of TB epidemiology, as incidence highlights prevention efforts while prevalence indicates the existing burden of the disease within a population [32,38,40].

### Disability Adjusted Life Years estimation

GBD computes DALY by combining the years of life lost (YLLs) and years lived with disability by illness (YLDs) due to TB. Thus, this composite measure reflects the whole burden of TB population health by combining fatal and non-fatal outcomes due to TB-related death and disability [37,38].

### Years of Life Lost (YLL) estimation

The YLLs measure the loss of life attributable to deaths due to TB by calculating the difference between life expectancy and age at death, multiplied by the total number of TB-related deaths [38]. It represents the burden of premature mortality caused by TB [41].

### Years Lived with Disability (YLD) estimation

The GBD study estimates YLDs to quantify the non-fatal health burden of TB by combining TB prevalence with its associated disability weights (DWs). YLDs are computed by multiplying the prevalence of TB cases and their respective DWs, which range from 0 to 1, where 0 means no loss in health due to TB and 1 means total loss of health (equivalent to death). Accounting for regional socioeconomic and varying impacts of TB on health, specific DWs were assigned to different forms of TB, including pulmonary TB (characterized by persistent cough, weight loss, and fatigue), TB meningitis (the most severe form, often leads to severe complications), and multi-drug resistant TB (MDR-TB), which requires prolonged and complicated treatment and has dire overall health risks). These measures are calculated from population-based studies through method logs such as paired comparisons or population health equivalence designed to measure societal preferences for different states of health [42]. Furthermore, all YLD estimates were lower and upper 95% UIs [38].

## Results

### Trends of TB incidence, prevalence, mortality and DALYs

The total new cases of TB in Eastern Africa were 679,627.4 (95% UI: 598,564.2, 766,373.7) with the prevalence of 93,858,669.2 (95% UI: 83,633,538.3, 106,060,151.9) in 2021. Total DALYs due to TB were 555,5004.2 (95% UI: 4,646,763.9, 6,675,264.6), and TB related deaths were 126,211.6 (95% UI: 104,359.5, 151,657.4) in Eastern Africa in 2021. In 1990, the age-standardized TB incidence rate was 518.8 per 100,000 (95% UI: 465.6–577.2), which dropped to 244.0 (95% UI: 217.2, 271.4) by 2021. This marks a 53% reduction in TB incidence between 1990 and 2021 (95% UI: −50.7%, −55.1%). Similarly, the prevalence of TB in Eastern Africa declined from 38,577.6 cases per 100,000 people (95% UI: 35,448.5 to 41,904.5) in 1990 and 27,366.1 per 100,000 (95% UI: 24,719.6, 30,228.2) in 2021, which is equivalent to a 29% reduction between 1990 and 2021 (95% UI: −26.3, −31.7). The decline in TB-related deaths from TB also dropped significantly from 191.5 deaths per 100,000 people (95% [UI]: 152.8, 233.9) in 1990 to 39.3 deaths per 100,000 (95% UI: 38.6, 39.9) in 2021. TB-related deaths decreased by 64.6% (95% [UI]: −55.0, −71.4) from 1990 to 2021. The overall disease burden, measured in DALYs, dropped from 6,709.2 DALYs per 100,000 people (95% UI: 5,560.8, 7,937.7) in 1990–2,132.9 DALYs per 100,000 (95% UI: 1,781.6, 2,534.3) in 2021, representing a 68.2% reduction (95% UI: −60.3, −73.6) from 1990 to 2021 “Table 1”.

**Table 1:** Trends in age standardized incidence, prevalence, mortality and DALYs of tuberculosis in Eastern Africa, 1990–2021.

Measure	Rates per 100, 000 population in Eastern Africa		Total percentage changes (%) from 1990 to 2021 (95% UI)
	1990 Estimate (95% UI)	2021 Estimate (95% UI)	
**Incidence**	518.8 (465.6, 577.2)	244.0 (217.2, 271.4)	−53.0(−50.7, −55.1)
**Prevalence**	38,577.6 (35,448.5, 41,904.5)	27,366.1 (24,719.6, 30,228.2)	−29.1 (−26.3 to −31.7)
**Death**	191.5 (152.8, 233.9)	67.9 (56.6, 81.2)	−60.6 (−55.0, −71.4)
**DALY**	6,709.2 (5,560.8, 7,937.7)	2,132.9 (1,781.6, 2,534.3)	−68.2 (−60.3, −73.6)

### TB incidence, prevalence, mortality and DALYs by age and sex

The burden of TB is disproportionately high among older adults across all indicators (incidence, prevalence, death, and DALY). The rate of TB incidence in children under 5 was 261.5 per 100,000 people (95% UI: 209.0, 324.6), and the prevalence was 21,596.6 per 100,000 (95% UI: 17,126.3, 26,187.7) in 1990. By 2021, both measures had significantly declined, with incidence dropping to 97.0 per 100,000 (95% UI: 75.1, 121.9) and prevalence decreasing to 12,603.6 per 100,000 (95% UI: 9,714.6, 15,839.0). Deaths from TB also dropped significantly, from 117.7 per 100,000 (95% UI: 92.6, 143.2) in 1990 to 19.4 per 100,000 (95% UI: 14.0, 26.5) in 2021. In the same way, DALYs saw a sharp decline from 10,632.2 per 100,000 (95% UI: 8,354.6, 12,837.2) in 1990–1,803.6 (95% UI: 1,325.1, 2,425.8) per 100,000 in 2021.

Among individuals aged 70 and above, TB rates remained substantially higher compared to younger populations. In this age group, the incidence rate was 1,514.3 (95% UI: 1,231.7, 1,807.5) per 100,000 in 1990, decreasing to 831.1 (95% UI: 662.4, 1,006.5) in 2021. Similarly, TB prevalence, mortality, and DALY declined, with prevalence decreasing from 50,785.8 per 100,000 in 1990–39,114.5 in 2021, mortality from 1,031.4 per 100,000 in 1990 to 467.3 in 2021, and DALYs dropping from 17,945.1 per 100,000 in 1990–7,729.1 in 2021.

Males were consistently more affected by TB than women. In 2021, the TB incidence rate among men was 278.4 (95% UI: 246.5, 308.8) per 100,000, compared to 213.0 per 100,000 (95% UI: 189.0, 237.1) among females. In 1990, the difference in TB incidence was not significantly different between males and females, but over time, males have shown a higher TB prevalence, mortality, and DALYs compared to females from 1990 to 2021. Overall, a decline has been observed in TB incidence, prevalence, mortality, and DALYs from 1990 to 2021 across all age groups and both sexes, but significant disparities exist between age and sex groups “Table 2”.

**Table 2:** TB incidence, prevalence, mortality and DALYs of Eastern Africa, 1990 and 2021 by age and sex.

Variable	Categories	Age standardized incidence rates per 100,000 population		Age standardized prevalence rates per 100,000 population		Age standardized death rate per 100,000 population		Age standardized DALYs due to TB, per 100,000 population	
		1990 Estimate (95% UI)	2021 Estimate (95% UI)	1990 Estimate (95% UI)	2021 Estimate (95% UI)	1990 Estimate (95% UI)	2021 Estimate (95% UI)	1990 Estimate (95% UI)	2021 Estimate (95% UI)
Age	<5years	261.5 (209.0, 324.6)	97.0 (75.1, 121.9)	21,596.6 (17,126.3, 26,187.7)	12,603.6 (9,714.6, 15,839.0)	117.7 (92.6, 143.2)	19.4 (14.0, 26.5)	10,632.2 (8,354.6, 12,837.2)	1,803.6 (1,325.1, 2,425.8)
	5-14	93.3 (65.2, 129.1)	44.0(28.8, 64.1)	25,832.4 (20,007.8, 32,833.2)	17,693.2 (12,874.7, 22,921.8)	6.4 (4.8, 7.9)	1.6(1.2, 1.9)	587.9 (462.5, 729.1)	155.1 (127.3, 188.3)
	15-49	388.5 (320.5, 466.1)	206.3 (171.2, 251.8)	41,484 (35,777.0, 46,867.8)	28,838.4 (24,576.4, 34,050.3)	65.9 (53.5, 79.3)	22.2 (17.8, 27.6)	3,855.6 (3,159.7, 4,614.4)	1,322.8 (1,081.1, 1,625.4)
	50-69	1,087.6 (862.2, 1,338.8)	431.2 (331.7, 540.1)	46,892.5 (39,951.5, 53,554.1)	35,730.4 (29,262.2, 41,836.4)	439.3 (355.2, 528.4)	134.9 (110.7, 162.9)	14,390.3 (11,824.7, 17,137.0)	4452.8 (3671.85, 5346.16)
	70 + years	1,514.3 (1,231.7, 1,807.5)	831.1 (662.4, 1,006.5)	50,785.8 (42,708.0, 57,530.8)	39,114.5 (31,932.7, 45,902.4)	1,031.4 (784.9, 1,308.1)	467.3 (388.1, 547.1)	17,945.1 (13,883.9, 22,455.2)	7,729.1 (6,505.3, 9,046.9)
Sex	Male	532.2 (475.3, 590.1)	278.4 (246.5, 308.8)	39,311.2 (36,132.0, 42,718.2)	28,671.1 (25,875.0, 31,722.6)	232.0 (162.3, 310.6)	89.8 (70.3, 109.4)	7,713.4 (5,537.7, 10,010.3)	2,763.7 (2,148.3, 3,395.0)
	Female	506.0(456.7, 565.8)	213.0 (189.0, 237.1)	37,870.1 (34,745.8, 41,155.6)	26,117.5 (23,507.7, 28,814.3)	152.3 (127.6, 191.8)	47.9 (41.1, 58.3)	5,734.0 (4,926.2, 6,939.3)	1,543.5 (1,311.1, 1,854.6)

### Age standardized TB incidence, prevalence, death and DALYs per 100,0000, across Eastern Africa countries

The incidence of TB per 100,000 people has declined across all countries in Eastern Africa. In 1990, Eritrea had the highest TB incidence in the region with 959.2 per 100,000 (95% UI: 868.7, 1,056.4). By 2021, Somalia had the highest incidence, with 658.7 per 100,000 (95% UI: 568.4, 753.7).

Mauritius consistently had the lowest TB incidence, with 22.7 per 100,000 (95% UI: 19.9, 25.8) in 1990 and 12.6 per 100,000 (11.1, 14.4) in 2021. Sudan had the third lowest TB incidence next to Mauritius and Seychelles and achieved the highest annual percentage decline of 3.2%. The TB incidence rate in Sudan dropped from 122.8 per 100,000 (95% UI: 111.0, 136.4) in 1990 to 47.7 (95% UI: 41.7, 54.5) in 2021 “Table 3”.

**Table 3:** Age-standardized incidence rates of TB in 1990 and 2021, and annual rate of changes across countries in Eastern Africa.

Countries	Age-standardized incidence rates per 100 000 population		Annual percent of change from 1990 to 2021
	1990 Estimate (95% UI)	2021 Estimate (95% UI)	
Comoros	536.9 (484.6, 590.2)	261.4 (226.3, 301.2)	−1.7
Djibouti	510.0 (465.0, 555.2)	249.6 (216.5, 283.9)	−2.0
Eritrea	959.2 (868.7, 1,056.4)	537.3 (470.7, 608.3)	−1.7
Ethiopia	750.8 (664.8, 848.1)	270.0(241.8, 297.3)	−3.0
Kenya	394.2 (345.6, 445.2)	240.1 (210.3, 268.1)	−1.2
Madagascar	619.4 (563.1, 683.4)	302.8 (265.2, 346.4)	−2.5
Mauritius	22.7 (19.9, 25.8)	12.6 (11.1, 14.4)	−1.0
Rwanda	536.4 (487.0, 612.8)	190.9 (167.0, 216.0)	−2.8
Seychelles	51.8 (45.6, 58.1)	30.9 (27.1, 34.9)	−1.0
Somalia	798.4 (702.1, 903.1)	658.7 (568.4, 753.7)	−1.1
South Sudan	593.1 (533.7, 660.8)	433.5 (381.3, 493.8)	−1.1
Sudan	122.8 (111.0, 136.4)	47.7 (41.7, 54.5)	−3.2
Uganda	386.0 (351.7, 420.1)	188.7 (163.2, 215.8)	−1.3
United Republic of Tanzania	458.8 (400.3, 522.4)	236.1 (214.6, 258.3)	−2.7

In the subnational assessment of Ethiopia in 2021, the highest TB incidence was recorded in the Afar (475.4 per 100,000; 95% UI: 401.3–553.8), Benishangul-Gumuz (400.8; 95% UI: 360.0–441.6), and Somalia (391.8; 95% UI: 353.6–429.0) regions. Somalia showed slower progress toward reducing the TB incidence from 1990 to 2021 when compared to other Ethiopian regions. Mandera in Kenya took the lead in recording the highest number of new TB cases in 2021 (622.6; 95% UI: 548.5–713.0), followed by Tana River (494.0; 95% UI: 439.3–553.4). On the other hand, TB incidence in Mandera has shown an increase in incidence over time. Dire Dawa (199.7; 95% UI: 175.9–225.5) and Harar (221.1; 95% UI: 197.2–246.9) in Ethiopia had the lowest TB incidence and also reported the highest average rate of annual reduction. In Kenya, Nakuru had the lowest TB incidence in 2021 (171.6; 95% UI: 148.6–194.8), followed by Bungoma (179.0; 95% UI: 155.3–201.1). The highest average annual rate of increase in TB incidence between 1990 and 2021 was recorded in Siaya “S1 Table”.

Between 1990 and 2021, the age-standardized prevalence of TB per 100,000 people dropped in most of the Eastern African countries, though the rate of reduction varied by country. Tanzania had the largest reduction in TB cases, with an average annual deduction of 2.4%. However, Tanzania had a high TB incidence in 2021 with 38,470.0 per 100,000 people (95% UI: 34,968.4, 42,339.5). In contrast, Mauritius was the only country in the region to experience a slight increase in TB cases, with an average annual rise of 0.1%. Every other country in the Eastern Africa region reduced TB cases during this period “Table 4”.

**Table 4:** Age-standardized prevalence rates of TB in 1990 and 2021, and annual rate of changes across countries in Eastern Africa.

Countries	Age-standardized prevalence rates 100, 000 population		Annual percent of change from 1990 to 2021
	1990 Estimate (95% UI)	2021 Estimate (95% UI)	
Comoros	40,323.0 (36,665.9, 44334.7)	23,173.1 (20,807.5,25,997.7)	−1.5
Djibouti	43,304.6 (40,178.9, 46,346.0)	20,215.2 (17,893.3, 22,686.8)	−2.3
Eritrea	44,248.6 (40,372.0, 48,067.7)	26,934.2 (23989.0, 30,329.8)	−1.6
Ethiopia	41,360.6 (37468.5, 45,459.5)	35,639.6 (32,303.7, 39,519.7)	−0.5
Kenya	31,348.1 (28,072.9, 34,865.6)	28,523.8 (25,413.8, 31,653.2)	−0.1
Madagascar	40,968.4 (37,172.3, 45,097.9)	24,745.2 (21,830.9, 27,928.9)	−1.7
Mauritius	30,979.0(27,495.2, 34,548.1)	28,067.4 (24,890.7, 31,532.6)	0.1
Rwanda	43,469.2 (39,828.9, 47,391.8)	22,551.7 (20,108.5, 25,329.8)	−2.1
Seychelles	33,572.5 (30,179.3, 37,291.6)	29,691.0 (26,424.4, 33,235.3)	−0.1
Somalia	33,761.2 (30,171.9, 37,429.4)	22,139.9 (19,740.8, 24,905.9)	−1.5
South Sudan	40,650.8 (36,876.9, 44,537.6)	24,846.5 (22,111.1, 27,892.3)	−1.7
Sudan	34,990.8 (32, 068.4, 38,106.5)	18,911.7 (16,594.7, 21,759.3)	−1.9
Uganda	51,080.0 (47,939.5, 53,864.1)	14,544.1 (12,932.6, 16,475.9)	−1.1
United Republic of Tanzania	31,974.1 (28,683.7, 35,572.1)	38,470.0 (34,968.4, 42,339.5)	−2.4

Having 40,066.7 cases per 100,000 population (95% UI: 36,228.3–43,878.7) in 2021, the Somalia region had the highest TB prevalence rate in Ethiopia. It is slightly nonevent that distinction over Benishangul-Gumuz with 39,969.5 cases (95% UI: 36,182.2–43,902.9). In contrast, Afar held the lowest recording with 29,606.7 (95% UI: 25,235.6–34,297.2) cases. Addis Ababa and Dire Dawa exhibited the highest average annual rate of change in TB prevalence across Ethiopian regions. Kenya, in 2021, saw Wajir accounting for the highest TB prevalence at 35,299.9 cases for 100,000 population (95% UI: 31,947.8–38,996.0), while West Pokot experienced the smallest with 25,113.4 cases (95% UI: 21,734.8–29,163.2) “S2 Table”.

The age-standardized TB-related mortality in Eastern African countries has shown a decreasing trend over time. Ethiopia had the highest TB-related mortality reduction, with mortality dropping from 348.3 per 100,000 (95% UI: 272.8, 413.5) in 1990 to 65.4 (95% UI: 64.85, 66.0) in 2021, reflecting an average annual percentage reduction of 6.0%. In 1990, Ethiopia had the highest TB-related mortality in the region, followed by Somalia. By 2021, Somalia had the highest TB-related mortality rate in the region, with a rate of 262.3 per 100,000 (95% UI: 253.1, 270.6). Kenya had the slowest reduction of TB-related mortality, with 0.9% “Table 5”.

**Table 5:** Age-standardized death rates of TB in 1990 and 2021, and annual rate of changes across countries in Eastern Africa.

Countries	Age-standardized death rates per 100, 000 population		Annual percent of change from 1990 to 2021
	1990 Estimate (95% UI)	2021 Estimate (95% UI)	
Comoros	178.1 (125.4, 239.4)	29.4 (28.9, 29.9)	−2.2
Djibouti	128.1 (88.3, 171.8)	25.3 (24.8, 25.8)	−1.9
Eritrea	280.4 (195.9, 371.3)	48.4 (47.7, 49.0)	−2.0
Ethiopia	348.3 (272.8, 413.5)	65.4 (64.9, 66.0)	−6.0
Kenya	140.6 (81.6, 225.2)	20.1 (19.7, 20.4)	−0.9
Madagascar	162.9 (125.8, 201.9)	50.0 (49.4, 50.7)	−2.9
Mauritius	3.7 (3.5, 3.9)	2.6 (2.5, 2.7)	−2.8
Rwanda	249.3 (175.5, 320.9)	39.3 (38.6, 39.9)	−4.9
Seychelles	9.4 (8.1, 10.5)	2.3 (2.2, 2.4)	−2.4
Somalia	296.1 (179.8, 499.4)	262.3 (253.1, 270.6)	−1.1
South Sudan	157.5 (104.0, 248.1)	107.8 (107.1, 108.5)	−1.4
Sudan	26.3 (16.6, 39.3)	17.8 (17.4, 18.1)	−5.6
Uganda	134.0 (91.6, 221.4)	35.3 (34.7, 35.9)	−2.9
United Republic of Tanzania	132.1 (96.6, 185.6)	42.5 (41.5, 42.7)	−3.4

In Ethiopia, in 2021, the Afar region had the highest rate of TB-related mortality at 155.0 deaths per 100,000 population (95% UI: 114.5–195.4), followed by the Somalia region at 121.3 (95% UI: 89.6–160.9). In contrast, Addis Ababa had the lowest TB-related death rate of 37.7 (95% UI: 28.7–49.8). In Kenya, Mandera had the maximum TB-related mortality as well, with rates pegged at 406.8 (95% UI: 192.6–615.5), followed by Wajir at 285.8 (95% UI: 144.5–459.0). The lowest TB-related death rate was recorded in Uasin Gishu (54.5; 95% UI: 30.1, 87.6). Wajir, Meru, Mandera, and Bomet observed an increasing trend in TB-related death incidence from 1990 to 2021 “S3 Table”.

There were significant disparities in DALY rates and trends among countries in Eastern Africa. In 1990, Ethiopia had the highest DALY in the region. However, Ethiopia also achieved the highest reduction rate in the region, from 11,895.4 per 100,000 (95% UI: 9,639.7, 13,811.9) in 1990–1,833.2 (95% UI: 1,559.8, 2,167.1) in 2021, with the average annual decline of 6.6%. In contrast, Somalia exhibited the highest DALY rate in 2021 in the region at 7,691.2 per 100,000 (95% UI: 4,664.6–13,131.8), with an average annual decline of just 1.42 “Table 6”.

**Table 6:** Age-standardized DALYs due to TB in 1990 and 2021, and annual rate of changes across countries in Eastern Africa.

Countries	Age-standardized DALYs rates per 100, 000 population		Annual percent of change from 1990 to 2021
	1990 Estimate (95% UI)	2021 Estimate (95% UI)	
Comoros	5,502.2(3,797.8, 7,443.9)	1,983.0 (1,440.9, 2667.3)	−3.0
Djibouti	4,374.2 (3,037.9, 5,944.3)	1,847.5 (1,094.6, 2,706.4)	−2.6
Eritrea	9,825.9 (6,958.4, 12,805.0)	4,645.7 (3,155.0, 7,158.2)	−2.4
Ethiopia	11,895.4 (9,639.7, 13,811.9)	1,833.2 (1,559.8, 2,167.1)	−6.6
Kenya	4,298.8 (2,578.3, 6,594.7)	2,795.5 (1,694.6, 3,743.5)	−1.5
Madagascar	5,989.9 (4,827.6, 7,309.2)	2,635.0 (1,926.9, 3,619.5)	−3.4
Mauritius	137.5 (127.4, 148.0)	39.5 (34.6, 44.6)	−2.8
Rwanda	8,443.6 (6,000.3, 10,681.7)	1,632.0 (1,085.2, 2,228.7)	−5.5
Seychelles	348.5 (305.8, 385.9)	127.7 (109.5, 150.2)	−2.3
Somalia	9,734.8 (6,130.3, 16,044.1)	7,691.2 (4,664.6, 13,131.8)	−1.4
South Sudan	5,623.5 (3,905.0, 8,433.7)	3,784.8 (2,655.4, 5,727.7)	−1.5
Sudan	1,002.8 (687.2, 1,424.5)	184.7 (116.8, 270.6)	−6.4
Uganda	4,367.1 (3,108.3, 6,952.0)	1,603.1 (1,138.3, 2,168.4)	−3.2
United Republic of Tanzania	4,639.9 (3,566.3, 6,278.6)	2,042.4 (1,548.7, 2,782.5)	−4.0

In Ethiopia, in 2021, the highest DALYs due to tuberculosis were recorded in Afar, with 4,451.5 DALYs per 100,000 population (95% UI: 3,354.4–5,549.9). On the contrary, Addis Ababa reported the lowest DALYs, at 1,202.6 (95% UI: 887.7–1,614.4). Dire Dawa and Harari recorded the highest increase in annual change of DALYs from 1990 to 2021. In Kenya, meanwhile, Bomet, Mandera, and Meru showed increases over the same period. Tana River reported the highest DALYs at 6,928.6 (95% UI: 4,246.3–9,860.1) in 2021, while Nakuru had the least at 1,748.3 (95% UI: 991.9–2,601.5) “S4 Table”.

## Discussion

Though, Eastern Africa has recorded a noticeable percentage reduction with respect to all metrics: incidence, prevalence, mortality, and DALYs, the burden of TB in the Eastern Africa region is still higher compared to global and other African countries. TB incidence and prevalence in Eastern Africa have decreased over time in terms of rates, while the absolute numbers have shown an increasing trend. This discrepancy may be due to the region’s rapid population growth. These countries include Ethiopia, Kenya, Uganda, and Tanzania, all of which have witnessed huge population changes over the last 30 years [43]. When a population grows at a faster rate, the total number of people who are susceptible to TB also rises [1,7].

Although, Tanzania was recorded to have experienced the highest annual percentage decline in TB prevalence, it has still the highest prevalence in 2021. The highest prevalence rate might be related to increase detection rates. A new TB detection technology were introduce in 2007. This was giant African pouched rates [44–46]. This comprehensive approaches might explain the stable annual decrease in TB prevalence. Ethiopia managed to show a passing decline in TB death. Indeed, the dipoles of the TB and HIV services in Ethiopia, integrated with complete healthcare interventions, may have led to a high reduction rate of death from TB [47,48]. Somalia in 2021 recorded the highest TB incidence rate in the entire Eastern Africa region and the TB-related DALYs with the slowest rate of decline. This large burden can be attributed to a fragile health system in the country that has been aggravated by prolonged periods of conflict, insecurity, low funding for TB programs, and poor implementation of global strategies for TB control [49,50].

Eastern Africa has had political instability and armed conflicts, setting back governance, development, and public health [51,52]. Conflict-affected areas experience disruption to health services, damage to infrastructure, and movement of populations, all compromising TB diagnosis, treatment, and follow-up [53,54]. Displacement increased the risk of contracting TB as a result of overcrowding, poor nutrition, and limited access to healthcare [52]. Often, in politically unstable settings, resources that are meant for healthcare are diverted to emergency or security needs, thereby further weakening the combating of TB through funding and implementation of programs [55]. In addition, economic hardship limits governments’ capacity to invest in the health system, contributing to poor living conditions and widespread malnutrition. It also continues to hinder households’ access to healthcare services, thereby increasing vulnerability to diseases such as tuberculosis. vulnerability to TB [56,57]. These structural barriers complicate attempts to ensure TB screening, diagnosis, and adherence to treatment [58].

In Eastern Africa, Eritrea was found to have the highest incidence of tuberculosis in the region in the year 1990, whereas in 2021, Somalia had the highest incidence. Conditions may be influenced by the lack of access to diagnosis, treatment, and follow-up care, which amplifies their conditions of problems associated with tuberculosis during this period [59]. The health infrastructure in Somalia is considered to be among the weakest across the region as well. Besides, both Eritrea and Somalia have suffered protracted conflicts, political instability, displacements, and overcrowding within living conditions, all adding up to poor access to healthcare and increased transmission of tuberculosis [49,60]. Eritrea was separated from Ethiopia in 1990, after which it had a health system that needed to be reorganized. The burden of TB in Mauritius was low in the eastern Africa region. Perhaps, such would include a strong health systems setting in Mauritius: universal health coverage with effective TB surveillance and a strong public health program that convergently contributes towards low TB incidence. High socioeconomic status, nutrition, and better living conditions help to reduce transmission and prevalence of TB [58,61]. Just immediately behind Mauritius and Seychelles, Sudan had the lowest incidence, and it showed a rapid increase in the annual percent change. Thus, the significant annual percent decline in TB incidence reflects great improvement in terms of TB programs such as DOTS, integrated TB-HIV services, and improved diagnostic capacities in the country [61,62].

Conversely, a large percentage reduction in TB incidence may be the result of TB control strategy in the region. The WHO formally launched the Directly Observed Treatment, Short-course (DOTS) strategy in 1994 as the standard global approach to TB control [63,64]. The introduction of the DOTS strategy in Eastern Africa during the 1990s had a substantial impact on tuberculosis control [65,66]. Standardized diagnosis and treatment, political commitment, uninterrupted drug supply, and robust monitoring and evaluation mechanisms were the main objectives of DOTS [64]. Within a decade of its implementation, many East African countries that adopted DOTS achieved treatment success rates exceeding 80% [65,67]. DOTS facilitated better integration of TB services into primary healthcare, and it gradually reduced TB incidence in the early 2000s [68]. However, the DOTS technical package improved overall treatment success but had no effect on case detection [69].

DOTS is not sufficient to achieve the 2015 tuberculosis-related Millennium Development Goals (MDG) [67]. While the Stop TB Strategy was aligned with the MDGs. The Stop TB initiative introduced a new approach that emphasized community-based interventions and the decentralization of TB care services [70,71]. These factors contributed to the success of controlling tuberculosis in Eastern Africa. As a result, the rates of incidence in Eastern Africa began to stabilize or decline modestly, and the treatment success rates improved in a few countries [72]. The subsequent integration of TB and HIV care was particularly successful in high-burden settings for reducing TB-related mortality in people living with HIV [73–75] (WHO, 2015; Stop TB Partnership, 2016). These achievements laid the foundation for the transition to the End TB Strategy under the Sustainable Development Goals (SDGs) [76].

The End TB Strategy, being SDG-aligned, has accelerated efforts to end and ultimately eliminate TB in the world, including Eastern Africa [76–78]. In the majority of the countries, the strategy has produced considerable progress by placing patient-centered care at the fore, reaching universal health coverage, making bold policy changes, and embracing new tools [79,80]. The use of e-health technologies, strong community involvement, and the decentralization of TB services have together strengthened case detection and boosted treatment adherence, especially in hard-to-reach areas [81]. However, challenges still exist in the regions, such as incompetent health systems and poverty. In essence, since the introduction of the End TB Strategy, Eastern Africa has made measurable strides toward reducing TB incidence and mortality; thus, the elimination targets by 2030 are quite ambitious and require sustained investments and innovations [1].

Mobile health and digital adherence tools were innovative technologies for TB diagnosis, treatment, and management [82–84]. Such resources improve the real-time communication, data monitoring, and patient care, resulting in improved case detection and treatment of TB [85]. Digital adherence technologies such as SMS reminders, video-observed therapy, and smart pillboxes increase the likelihood that patients will take medications as directed, thereby decreasing the probability of treatment failure or drug resistance TB [86,87]. Additionally, electronic medical records systems and mobile technology-based systems of data collection can help with timely reporting and surveillance such that health systems can track the trends and respond more appropriately [83,85]. Together, these technology solutions bridge current gaps in the TB care cascade and enhance the pace of progress toward TB elimination targets [88].

Community-based interventions and public awareness initiatives have been essential in enhancing TB prevention and treatment adherence [89,90]. They have supported bridging gaps concerning healthcare provision. Local health workers, volunteers, and community leaders could be engaged in providing education on TB, early case identification of suspects, and observation and support for completing treatment [91,92]. In Eastern Africa, countries had community-based interventions and public awareness campaigns for TB control and prevention [93]. For instance, Ethiopia has successfully utilized health extension programs, and Uganda has used village health teams [94–96].Funding and resource allocation are key factors in determining the effectiveness of TB control programs [97]. The Eastern African region has continued to face barriers in generating and sustaining sufficient resources to fund TB prevention, diagnosis, treatment, and monitoring [72]. Despite some states’ political commitments, most countries in this region continue to heavily rely on external donors. For instance, in 2020, some 60% of the TB finance in the major source countries of Ethiopia, Tanzania, Kenya, and Uganda still came from outside, with contributions from within those countries only barely meeting a fraction of their need [98,99]. Vulnerabilities risk being introduced through the attitude of over-dependence upon support donor priorities shift or funding diminishes [100]. Thus, to improve TB outcomes in Eastern Africa countries, resource mobilization has to be enhanced domestically; health financing systems have to be strengthened; and TB has to be prioritized in national health budgets.

The emergence of multidrug-resistant tuberculosis (MDR-TB) has brought about a considerable setback in tuberculosis control in the region [101]. Treatment of MDR-TB poses a significant threat to TB control efforts because of its complexity and costs for treatment, long treatment duration, lower treatment success rates, and, principally, increased risk of death as compared to drug-susceptible TB [102,103]. Secondly, the disease places an enormous socioeconomic strain on health systems and patients, with prolonged inpatient care, socioeconomic difficulties, and stigma [104–106].

In the results of this study, older adults are more susceptible to TB incidence, most likely due to an age-related decline in immune function. Thus, the effect of weakened immunity leaves them susceptible to TB infection, with possible progression to the active disease stage. Earlier in life, TB infected many older adults, and they may now harbor latent TB infections [107]. As immunity wanes with age, these latent infections can reactivate, and thus, more TB patients would be grouped under an older age category, which has higher prevalence and mortality [108]. Apart from the above, other common comorbidities among old-age persons also make them more susceptible to TB and lead to a deterioration of outcomes. Symptoms of TB in older adults are frequently similar to those of other chronic diseases, complicating their diagnosis and treatment. The result is worsened outcomes, especially concerning increased mortality and DALYs [109].

The study reveals that, the incidence of TB for men is higher than for females. The reason for this discrepancy may be due to the differences in immune response. Estrogen is thought to provide some protective effects against TB in women, while testosterone may increase susceptibility [110]. Additionally, men are more likely to engage in behaviors that increase TB risk, such as smoking, alcohol consumption, and working in environments with higher TB exposure [111,112]. In certain situations, gender and cultural norms may lead to underreporting or underdiagnosis of TB in women perturbed enough to have similar or even higher rates of infection. This can further exaggerate the apparent discrepancy in the incidence of TB between men and women [113]. As in the case of incidence, there was a marked difference between men and women in terms of the prevalence of TB, TB-related deaths, and DALYs. This can be attributed to biology, behavior, and social factors [58,110,112]. Men mostly work in high-risk environments for TB exposure, such as mines and crowded workplaces, particularly in low-income areas [114].

### Policy implications

The study identifies the important areas for tuberculosis control in Eastern Africa and recommends policy improvements that will ensure better prevention, diagnosis, treatment, and management. Key recommendations include the strengthening of TB control programs, health infrastructure, social determinants of health, more health care financing, developing conflict-sensitive TB strategies, utilizing technology for TB control, and maintaining global partnerships with multisectoral collaborations. The need for more funding of healthcare, increased community engagement, gender-sensitive TB interventions, and multisectoral coordination of governments with NGOs, research institutions, and international health organizations calls for more concentrated effort in this region. In many ways, it can be a pathway to how Eastern Africa should rev up its efforts towards TB reduction in meeting the global elimination targets of the World Health Organization and Sustainable Development Goals.

### Strength and limitations of the study

The study investigates the burden of TB in Eastern Africa, describing the incidence, prevalence, mortality, and DALYs trends over the years. It further goes on to make a comparison with global averages, highlighting regional inequities and trends. The findings have relevance for public health policy and strategies with a view to accelerating the reduction of TB burdens and the achievement of global TB elimination targets. The geopolitical instability and socioeconomic elements are hard to capture and may affect health care and efforts for TB control. In this study, subnational data is not available that may influence the effectiveness of TB control programs and health outcomes distribution in the region.

## Conclusion

The burden of TB in Eastern Africa has significantly declined over the past three decades. Whereas, the weight of TB in Eastern Africa is still greater than the global and African averages. The incidence, prevalence, mortality, and DALYs due to TB for the period 1990–2021 have displayed declining tendencies in the region. Old persons suffer more from TB diseases and TB outcomes than the younger populations. More men are burdened by TB than women. In 1990, the highest rate of incidence was reported in Eritrea as compared to other Eastern Africa countries. Ethiopia, showed the greatest decrease in tuberculosis-related deaths and DALYs. Conversely, the other countries with low tuberculosis incidence rates were Sudan, Mauritius, and Seychelles. Additionally, within the region, Somalia had high incidences prevalence, mortality, and DALY figures. The study recommends policy improvements in Eastern Africa for tuberculosis control, including strengthening control programs, health infrastructure, social determinants, healthcare financing, conflict-sensitive strategies, technology use, and global partnerships.

## Supporting information

S1 Table Subnational Age-standardized incidence rates of TB in 1990 and 2021, and annual rate of changes in Ethiopia and Kenya. (DOCX)

S2 Table Subnational Age-standardized prevalence rates of TB in 1990 and 2021, and annual rate of changes in Ethiopia and Kenya. (DOCX)

S3 Table Subnational Age-standardized death rates of TB in 1990 and 2021, and annual rate of changes in Ethiopia and Kenya. (DOCX)

S4 Table Subnational Age-standardized DALYs due to TB in 1990 and 2021, and annual rate of changes in Ethiopia and Kenya. (DOCX)

## Abbreviations

CODEm: Cause of Death Ensemble Modeling
DALY: Disability-Adjusted Life Year
DOTS: Directly Observed Treatment, Short-course
GBD: Global Burden of Disease
DWs: Disability weights
ICD: Classification of Diseases
IHME: Institute for Health Metrics and Evaluation
MDG: Millennium Development Goals
MDR-TB: Multidrug-resistant tuberculosis
SDG: Susceptible Development Goal
TB: Tuberculosis
UIs: Uncertainty Intervals
UN: United Nations
WHO: World Health Organization
YLLs: Years of life lost
