# Supplementary material for: Burden of tuberculosis in Eastern Africa region from 1990–2021: A systematic analysis for the Global Burden of Disease 2021 Study
Source: PLoS One. 2025 Sep 2;20(9):e0331035. doi: 10.1371/journal.pone.0331035 (PMC12404479; doi:10.1371/journal.pone.0331035)
Supplement: S1 Table — (DOCX) [file pone.0331035.s001.docx]

S1 Table. Subnational Age-standardized incidence rates of TB in 1990 and 2021, and annual rate of changes in Ethiopia and Kenya

| **Country** | **Subnational group** | **Age-standardized DALYs rates per 100, 000 population** | | **Annual percent of change from 1990 to 2021** |
| --- | --- | --- | --- | --- |
|  |  | **1990 Estimate (95% UI)** | **2021 Estimate (95% UI)** |  |
| **Ethiopia** | Addis Ababa | 764.7 (683.5, 861.1) | 241.1 (212.6, 274.9) | -3.7 |
|  | Afar | 1140.2 (951.1, 1355.3) | 475.4 (401.3, 553.8) | -2.8 |
|  | Amhara | 716.6 (644.0, 796.6) | 257.2 (229.4, 284.8) | -3.3 |
|  | Benishangul-Gumuz | 1183.9 (1058.0, 1323.2) | 400.8 (360.0, 441.6) | -3.5 |
|  | Dire Dawa | 725.5 (652.2, 810.9) | 199.7 (175.9, 225.5) | -4.2 |
|  | Gambella | 841.9 (758.6, 942.3) | 258.7 (228.1, 288.8) | -3.8 |
|  | Harari | 799.4 (712.9, 887.1) | 221.1 (197.2, 246.9) | -4.2 |
|  | Oromia | 771.9 (655.2, 901.3) | 253.0 (223.7, 279.9) | -3.6 |
|  | Somali | 752.6 (672.5, 844.3) | 391.8 (353.6, 429.0) | -2.1 |
|  | Southern Nations, Nationalities, and Peoples | 722.5 (643.4, 800.1) | 281.3 (255.3, 308.5) | -3.0 |
|  | Tigray | 821.9 (725.3, 932.5) | 257.7 (229.9, 286.1) | -3.7 |
| **Kenya** | Baringo | 465.4 (413.5, 520.1) | 258.4 (224.3, 289.4) | -1.9 |
|  | Bomet | 258.6 (222.7, 304.1) | 193.0 (167.5, 219.7) | -0.9 |
|  | Bungoma | 369.2 (321.9, 418.0) | 179.0 (155.3, 201.1) | -2.3 |
|  | Busia | 426.7 (369.1, 488.5) | 228.6 (198.2, 255.5) | -2.0 |
|  | Elgeyo-Marakwet | 454.3 (399.5, 510.9) | 254.4 (222.9, 286.5) | -1.9 |
|  | Embu | 316.5 (275.2, 362.4) | 206.7 (180.3, 234.4) | -1.4 |
|  | Garissa | 623.7 (550.9, 704.9) | 465.4 (408.1, 520.1) | -0.9 |
|  | Homa Bay | 527.2 (458.7, 606.3) | 278.9 (239.9, 316.5) | -2.1 |
|  | Isiolo | 606.0 (543.4, 674.4) | 345.6 (305.1, 388.0) | -1.8 |
|  | Kajiado | 373.1 (326.0, 424.4) | 208.6 (181.7, 235.0) | -1.9 |
|  | Kakamega | 427.9 (374.4, 483.9) | 245.4 (214.7, 277.7) | -1.8 |
|  | Kericho | 359.8 (311.3, 414.1) | 181.2 (157.2, 206.2) | -2.2 |
|  | Kiambu | 404.4 (352.0, 457.8) | 229.8 (200.8, 260.9) | -1.8 |
|  | Kilifi | 423.2 (372.6, 480.1) | 251.8 (220.5, 285.1) | -1.7 |
|  | Kirinyaga | 328.1 (284.5, 377.1) | 221.8 (193.5, 251.1) | -1.3 |
|  | Kisii | 468.9 (416.1, 523.0) | 272.9 (237.0, 306.7) | -1.8 |
|  | Kisumu | 485.5 (417.8, 563.7) | 280.3 (246.7, 320.2) | -1.8 |
|  | Kitui | 421.8 (365.7, 483.2) | 247.5 (216.5, 278.4) | -1.7 |
|  | Kwale | 461.2 (406.2, 519.8) | 287.5 (253.1, 321.8) | -1.5 |
|  | Laikipia | 340.4 (293.0, 390.6) | 189.4 (163.9, 216.5) | -1.9 |
|  | Lamu | 433.7 (382.4, 490.9) | 274.2 (240.1, 304.7) | -1.5 |
|  | Machakos | 305.3 (262.4, 350.9) | 195.8 (170.3, 221.1) | -1.4 |
|  | Makueni | 370.9 (322.9, 425.7) | 216.9 (189.8, 246.3) | -1.7 |
|  | Mandera | 608.2 (537.1, 686.9) | 622.6 (548.5, 713.0) | 0.1 |
|  | Marsabit | 493.7 (434.9, 559.5) | 351.8 (306.7, 396.2) | -1.1 |
|  | Meru | 342.7 (297.1, 392.2) | 263.7 (231.9, 294.7) | -0.8 |
|  | Migori | 486.5 (428.8, 545.4) | 287.3 (251.2, 324.2) | -1.7 |
|  | Mombasa | 338.2 (293.3, 383.9) | 195.6 (170.0, 221.8) | -1.8 |
|  | Murang'a | 326.8 (282.0, 372.5) | 218.4 (189.9, 249.1) | -1.3 |
|  | Nairobi | 295.6 (257.8, 336.8) | 179.1 (155.5, 204.4) | -1.6 |
|  | Nakuru | 344.8 (299.3, 394.8) | 171.6 (148.6, 194.8) | -2.3 |
|  | Nandi | 429.7 (372.7, 485.6) | 233.0 (202.7, 263.6) | -2.0 |
|  | Narok | 436.5 (386.0, 487.8) | 301.0 (264.8, 339.8) | -1.2 |
|  | Nyamira | 429.4 (371.4, 490.1) | 329.0 (289.0, 366.8) | -0.9 |
|  | Nyandarua | 402.5 (352.5, 453.3) | 307.7 (268.6, 344.3) | -0.9 |
|  | Nyeri | 378.3 (330.9, 430.4) | 243.1 (213.4, 272.5) | -1.4 |
|  | Samburu | 420.8 (363.9, 486.1) | 304.5 (263.7, 347.8) | -1.0 |
|  | Siaya | 517.0 (446.3, 596.4) | 224.9 (200.8, 249.8) | -2.7 |
|  | Taita Taveta | 347.5 (301.7, 395.6) | 271.2 (239.9, 303.7) | -0.8 |
|  | Tana River | 649.5 (568.6, 724.9) | 494.0 (439.3, 553.4) | -0.9 |
|  | Tharaka Nithi | 437.1 (384.1, 495.5) | 231.7 (203.4, 259.9) | -2.1 |
|  | Trans Nzoia | 322.9 (280.0, 372.5) | 194.5 (169.7, 221.3) | -1.6 |
|  | Turkana | 553.1 (489.3, 622.6) | 400.2 (350.2, 453.1) | -1.0 |
|  | Uasin Gishu | 298.6 (258.7, 341.3) | 165.1 (143.0, 187.0) | -1.9 |
|  | Vihiga | 402.4 (352.4, 460.1) | 253.4 (222.6, 284.7) | -1.5 |
|  | Wajir | 483.7 (426.5, 547.6) | 454.2 (405.8, 507.0) | -0.2 |
|  | West Pokot | 466.3 (410.5, 524.9) | 287.5 (251.7, 321.6) | -1.6 |
