# Supplementary material for: Burden of tuberculosis in Eastern Africa region from 1990–2021: A systematic analysis for the Global Burden of Disease 2021 Study
Source: PLoS One. 2025 Sep 2;20(9):e0331035. doi: 10.1371/journal.pone.0331035 (PMC12404479; doi:10.1371/journal.pone.0331035)
Supplement: S2 Table — (DOCX) [file pone.0331035.s002.docx]

S2 Table. Subnational Age-standardized prevalence rates of TB in 1990 and 2021, and annual rate of changes in Ethiopia and Kenya

| **Country** | **Subnational group** | **Age-standardized DALYs rates per 100, 000 population** | | **Annual percent of change from 1990 to 2021** |
| --- | --- | --- | --- | --- |
|  |  | **1990 Estimate (95% UI)** | **2021 Estimate (95% UI)** |  |
| **Ethiopia** | Addis Ababa | 41977.1 (38289.9, 46328.9) | 30417.1 (27207.5, 34221.2) | -1.0 |
|  | Afar | 34704.9 (29503.8, 40339.0) | 29606.7 (25235.6, 34297.2) | -0.5 |
|  | Amhara | 44681.2 (40687.3, 48605.0) | 36036.1 (32563.0, 40106.9) | -0.7 |
|  | Benishangul-Gumuz | 47190.5 (42864.4, 51407.2) | 39969.5 (36182.2, 43902.9) | -0.5 |
|  | Dire Dawa | 44345.1 (40474.7, 48402.8) | 33029.9 (29369.4, 36782.9) | -1.0 |
|  | Gambella | 45219.8 (41457.4, 49333.3) | 34945.8 (31427.5, 38809.7) | -0.8 |
|  | Harari | 45486.5 (41245.7, 49697.6) | 34356.4 (30593.6, 38186.2) | -0.9 |
|  | Oromia | 34674.9 (29761.7, 39760.0) | 34136.1 (30656.9, 38054.1) | -0.1 |
|  | Somali | 42938.1 (39123.0, 47292.3) | 40066.7 (36228.3, 43878.7) | -0.2 |
|  | Southern Nations, Nationalities, and Peoples | 45674.7 (41390.6, 49861.9) | 38030.5 (34402.1, 42155.2) | -0.6 |
|  | Tigray | 44921.8 (40941.9, 49048.4) | 35032.0 (31533.1, 39058.4) | -0.8 |
| **Kenya** | Baringo | 36506.3 (32871.2, 40491.0) | 31357.9 (27818.3, 27818.3) | -0.5 |
|  | Bomet | 25446.9 (22513.8, 28925.7) | 24083.7 (21430.1, 21430.1) | -0.2 |
|  | Bungoma | 32666.6 (29103.5, 36519.6) | 27725.0 (24639.8, 24639.8) | -0.5 |
|  | Busia | 32834.9 (29190.6, 36753.6) | 29299.0 (25997.0, 25997.0) | -0.4 |
|  | Elgeyo-Marakwet | 33969.4 (30587.3, 37831.5) | 29755.7 (26404.6, 26404.6) | -0.4 |
|  | Embu | 28749.5 (25486.5, 32127.5) | 27015.7 (23941.5, 23941.5) | -0.2 |
|  | Garissa | 34415.6 (30890.3, 38458.4) | 32805.0 (29300.8, 29300.8) | -0.2 |
|  | Homa Bay | 35300.3 (31536.5, 39222.1) | 29154.3 (25900.0, 25900.0) | -0.6 |
|  | Isiolo | 37317.2 (33807.8, 41390.5) | 32776.6 (29489.3, 29489.3) | -0.4 |
|  | Kajiado | 31550.2 (28092.9, 34983.1) | 27428.7 (24265.2, 24265.2) | -0.5 |
|  | Kakamega | 33887.6 (30174.8, 37888.1) | 29012.4 (25756.5, 25756.5) | -0.5 |
|  | Kericho | 28386.4 (25239.0, 31755.4) | 26260.3 (23112.7, 23112.7) | -0.3 |
|  | Kiambu | 31329.1 (27799.0, 35378.3) | 28676.7 (25429.0, 25429.0) | 0.1 |
|  | Kilifi | 32480.0 (28980.3, 36335.0) | 28779.4 (25215.1, 25215.1) | -0.4 |
|  | Kirinyaga | 29523.5 (26186.8, 33198.4) | 27745.2 (24421.9, 24421.9) | -0.2 |
|  | Kisii | 35586.3 (31985.0, 39254.0) | 30105.7 (26960.4, 26960.4) | -0.5 |
|  | Kisumu | 30682.4 (27092.3, 34563.6) | 26803.9 (23880.4, 23880.4) | -0.4 |
|  | Kitui | 32119.8 (28711.4, 35915.4) | 27857.5 (24772.0, 24772.0) | -0.5 |
|  | Kwale | 33772.7 (30354.3, 37712.9) | 30574.8 (27165.3, 27165.3) | -0.3 |
|  | Laikipia | 27454.0 (24362.6, 30831.2) | 24558.0 (21915.3, 21915.3) | -0.4 |
|  | Lamu | 32483.4 (28848.0, 36557.1) | 30081.8 (26703.3, 26703.3) | -0.3 |
|  | Machakos | 28661.2 (25513.5, 32017.4) | 27118.5 (23885.3, 23885.3) | -0.2 |
|  | Makueni | 30119.0 (26793.3, 33665.5) | 25039.3 (22181.2, 22181.2) | -0.6 |
|  | Mandera | 35015.7 (31307.5, 39406.8) | 34603.3 (31216.3, 31216.3) | -0.1 |
|  | Marsabit | 32855.0 (29354.0, 36729.2) | 30917.1 (27596.7, 27596.7) | -0.2 |
|  | Meru | 29175.4 (25780.6, 32846.4) | 29318.1 (26074.7, 26074.7) | 0.1 |
|  | Migori | 35616.4 (31927.3, 39261.5) | 31613.8 (28030.1, 28030.1) | -0.4 |
|  | Mombasa | 28772.6 (25494.0, 32023.4) | 26828.1 (23695.6, 23695.6) | -0.2 |
|  | Murang'a | 27928.8 (24816.0, 31312.5) | 26056.5 (23220.5, 23220.5) | -0.2 |
|  | Nairobi | 29354.0 (26060.8, 32829.5) | 26620.3 (23353.5, 23353.5) | -0.3 |
|  | Nakuru | 29863.8 (26433.5, 33144.5) | 27035.7 (23904.1, 23904.1) | -0.3 |
|  | Nandi | 32236.8 (28652.1, 35910.5) | 27649.7 (24528.9, 24528.9) | -0.5 |
|  | Narok | 34827.2 (31438.4, 38899.3) | 30921.1 (27592.1, 27592.1) | -0.4 |
|  | Nyamira | 31615.0 (28246.5, 35576.9) | 31717.2 (28227.4, 28227.4) | 0.1 |
|  | Nyandarua | 31451.0 (27979.0, 34815.3) | 31226.8 (27860.6, 27860.6) | -0.1 |
|  | Nyeri | 30118.7 (26751.9, 33395.0) | 28207.0 (24972.0, 24972.0) | -0.2 |
|  | Samburu | 30758.4 (27454.9, 34228.7) | 29755.3 (26345.1, 26345.1) | -0.1 |
|  | Siaya | 32016.8 (27992.5, 35948.5) | 33111.0 (29838.0, 29838.0) | 0.1 |
|  | Taita Taveta | 31047.1 (27796.0, 34466.4) | 29696.0 (26460.7, 26460.7) | -0.1 |
|  | Tana River | 35299.9 (31740.4, 39127.9) | 34301.3 (31072.7, 31072.7) | -0.1 |
|  | Tharaka Nithi | 33961.5 (30429.7, 37800.1) | 30664.4 (27300.1, 27300.1) | -0.3 |
|  | Trans Nzoia | 28230.0 (25016.1, 31608.9) | 25271.1 (22396.9, 22396.9) | -0.4 |
|  | Turkana | 33522.7 (29869.6, 37669.9) | 30065.7 (26569.4, 26569.4) | -0.4 |
|  | Uasin Gishu | 28513.2 (25269.3, 32039.6) | 25570.3 (22746.3, 22746.3) | -0.4 |
|  | Vihiga | 32995.7 (29546.2, 36677.3) | 30182.7 (26712.2, 26712.2) | -0.3 |
|  | Wajir | 36683.4 (33098.3, 40812.2) | 35299.9 (31947.8, 31947.8) | -0.1 |
|  | West Pokot | 28389.2 (24822.5, 32461.0) | 25113.4 (21734.8, 29163.2) | -0.4 |
