# Supplementary material for: Burden of tuberculosis in Eastern Africa region from 1990–2021: A systematic analysis for the Global Burden of Disease 2021 Study
Source: PLoS One. 2025 Sep 2;20(9):e0331035. doi: 10.1371/journal.pone.0331035 (PMC12404479; doi:10.1371/journal.pone.0331035)
Supplement: S3 Table — (DOCX) [file pone.0331035.s003.docx]

S3 Table. Subnational Age-standardized death rates of TB in 1990 and 2021, and annual rate of changes in Ethiopia and Kenya

| **Country** | **Subnational group** | **Age-standardized DALYs rates per 100, 000 population** | | **Annual percent of change from 1990 to 2021** |
| --- | --- | --- | --- | --- |
|  |  | **1990 Estimate (95% UI)** | **2021 Estimate (95% UI)** |  |
| **Ethiopia** | Addis Ababa | 252.9 (183.0, 308.7) | 37.7 (28.7, 49.8) | -6.1 |
|  | Afar | 722.0 (520.8, 905.7) | 155.0 (114.5, 195.4) | -5.0 |
|  | Amhara | 306.1 (230.9, 383.2) | 55.3 (43.3, 69.5) | -5.5 |
|  | Benishangul-Gumuz | 661.4 (491.0, 838.5) | 113.2 (88.8, 143.1) | -5.7 |
|  | Dire Dawa | 305.7 (219.9, 384.8) | 41.4 (28.6, 63.6) | -6.5 |
|  | Gambella | 393.0 (266.7, 502.3) | 61.5 (42.0, 86.8) | -6.0 |
|  | Harari | 366.8 (261.0, 448.1) | 46.7 (31.3, 68.8) | -6.7 |
|  | Oromia | 392.1 (295.4, 483.8) | 56.9 (46.7, 69.2) | -6.2 |
|  | Somali | 308.1 (213.2, 477.8) | 121.3 (89.6, 160.9) | -3.0 |
|  | Southern Nations, Nationalities, and Peoples | 345.4 (263.4, 421.4) | 67.6 (52.5, 86.4) | -5.3 |
|  | Tigray | 384.7 (286.1, 467.4) | 57.5 (44.4, 74.5) | -6.1 |
| **Kenya** | Baringo | 229.6 (122.3, 348.9) | 120.6 (70.0, 178.4) | -2.1 |
|  | Bomet | 60.4 (25.9, 136.8) | 65.8 (26.9, 110.6) | 0.3 |
|  | Bungoma | 130.2 (68.7, 215.8) | 60.3 (36.2, 86.9) | -2.5 |
|  | Busia | 156.6 (76.2, 279.9) | 84.1 (47.0, 131.4) | -2.0 |
|  | Elgeyo-Marakwet | 171.9 (88.9, 286.2) | 87.9 (52.9, 140.7) | -2.2 |
|  | Embu | 84.7 (44.5, 159.2) | 67.3 (35.6, 111.0) | -0.7 |
|  | Garissa | 265.3 (143.7, 455.0) | 223.9 (122.7, 366.6) | -0.6 |
|  | Homa Bay | 187.9 (100.3, 298.2) | 105.4 (54.8, 160.9) | -1.9 |
|  | Isiolo | 255.3 (143.2, 380.3) | 143.3 (80.7, 216.4) | -1.9 |
|  | Kajiado | 129.3 (62.8, 240.1) | 81.3 (41.2, 131.8) | -1.5 |
|  | Kakamega | 171.2 (93.1, 259.6) | 104.5 (59.9, 152.9) | -1.6 |
|  | Kericho | 103.7 (45.0, 220.6) | 55.4 (31.3, 94.4) | -2.0 |
|  | Kiambu | 165.1 (91.2, 247.7) | 102.8 (53.7, 141.4) | -1.5 |
|  | Kilifi | 137.4 (70.5, 246.1) | 89.5 (53.6, 132.7) | -1.4 |
|  | Kirinyaga | 104.9 (53.3, 201.3) | 83.4 (41.7, 124.5) | -0.7 |
|  | Kisii | 212.4 (116.9, 305.7) | 130.9 (77.5, 181.2) | -1.6 |
|  | Kisumu | 189.4 (92.9, 319.4) | 118.4 (53.3, 172.3) | -1.5 |
|  | Kitui | 122.8 (66.0, 217.0) | 83.2 (42.8, 134.2) | -1.3 |
|  | Kwale | 159.4 (87.0, 278.7) | 115.3 (64.3, 175.8) | -1.0 |
|  | Laikipia | 97.2 (49.4, 195.2) | 57.2 (28.2, 92.9) | -1.7 |
|  | Lamu | 153.9 (81.6, 264.9) | 111.6 (62.6, 183.5) | -1.4 |
|  | Machakos | 81.3 (39.3, 157.8) | 66.2 (37.8, 102.3) | -0.7 |
|  | Makueni | 91.3 (47.6, 165.0) | 63.1 (29.5, 109.9) | -1.2 |
|  | Mandera | 255.7 (126.5, 454.1) | 406.8 (192.6, 615.5) | 1.5 |
|  | Marsabit | 163.6 (78.2, 294.7) | 142.4 (69.1, 241.7) | -0.5 |
|  | Meru | 101.1 (50.1, 192.4) | 101.6 (57.3, 158.8) | 0.1 |
|  | Migori | 183.3 (97.3, 293.8) | 122.1 (66.5, 184.8) | -1.3 |
|  | Mombasa | 122.1 (61.1, 217.3) | 77.8 (40.8, 116.3) | -1.5 |
|  | Murang'a | 102.5 (50.3, 188.0) | 80.8 (38.2, 116.0) | -0.8 |
|  | Nairobi | 121.6 (61.8, 179.9) | 83.3 (38.5, 116.4) | -1.2 |
|  | Nakuru | 124.3 (58.7, 229.2) | 64.0 (34.6, 98.5) | -2.1 |
|  | Nandi | 160.2 (80.4, 286.9) | 91.1 (44.5, 137.8) | -1.8 |
|  | Narok | 196.4 (93.7, 347.6) | 157.5 (83.4, 232.9) | -1.7 |
|  | Nyamira | 173.9 (94.2, 272.2) | 167.8 (99.2, 234.0) | -1.1 |
|  | Nyandarua | 178.8 (101.9, 274.1) | 159.9 (93.2, 220.7) | -0.4 |
|  | Nyeri | 126.2 (70.4, 205.3) | 96.9 (56.6, 134.9) | -0.9 |
|  | Samburu | 139.7 (63.1, 257.7) | 132.1 (62.9, 240.3) | -0.2 |
|  | Siaya | 192.0 (99.4, 315.3) | 65.5 (35.9, 105.3) | -3.5 |
|  | Taita Taveta | 118.0 (61.7, 229.0) | 110.1 (62.8, 163.2) | -0.2 |
|  | Tana River | 269.8 (150.2, 459.6) | 245.5 (149.5, 351.7) | -0.3 |
|  | Tharaka Nithi | 196.8 (113.8, 306.2) | 74.8 (44.9, 108.8) | -3.1 |
|  | Trans Nzoia | 100.5 (45.5, 203.3) | 69.1 (33.1, 110.1) | -1.2 |
|  | Turkana | 183.8 (80.0, 338.6) | 166.2 (79.9, 309.9) | -0.3 |
|  | Uasin Gishu | 89.3 (45.1, 185.6) | 54.5 (30.1, 87.6) | -1.6 |
|  | Vihiga | 130.5 (75.5, 206.0) | 100.7 (63.0, 144.8) | -0.8 |
|  | Wajir | 274.4 (128.4, 454.3) | 285.8 (144.5, 459.0) | 0.1 |
|  | West Pokot | 179.3 (92.3, 306.9) | 119.7 (69.7, 197.7) | -1.3 |
