# Supplementary material for: Burden of tuberculosis in Eastern Africa region from 1990–2021: A systematic analysis for the Global Burden of Disease 2021 Study
Source: PLoS One. 2025 Sep 2;20(9):e0331035. doi: 10.1371/journal.pone.0331035 (PMC12404479; doi:10.1371/journal.pone.0331035)
Supplement: S4 Table — (DOCX) [file pone.0331035.s004.docx]

S4 Table. Subnational Age-standardized DALYs due to TB in 1990 and 2021, and annual rate of changes in Ethiopia and Kenya

| **Country** | **Subnational group** | **Age-standardized DALYs rates per 100, 000 population** | | **Annual percent of change from 1990 to 2021** |
| --- | --- | --- | --- | --- |
|  |  | **1990 Estimate (95% UI)** | **2021 Estimate (95% UI)** |  |
| **Ethiopia** | Addis Ababa | 8,669.3 (6,336.8, 10,602.3) | 1,202.6 (887.7, 1,614.4) | -6.4 |
|  | Afar | 2,6656.9 (20,246.0, 32,970.5) | 4,451.5 (3,354.4, 5,549.9) | -5.8 |
|  | Amhara | 10,535.4 (8,365.0, 12,919.6) | 1,617.2 (1,284.4, 2,005.3) | -6.1 |
|  | Benishangul-Gumuz | 24,147.5 (18,734.4, 30,229.2) | 3,436.0 (2,774.6, 4,233.1) | -6.3 |
|  | Dire Dawa | 11,172.4 (7,970.8, 13,975.0) | 1,155.4 (805.2, 1,723.9) | -7.3 |
|  | Gambella | 14,728.3 (10,748.3, 18,258.1) | 1,775.9 (1,263.1, 2,506.6) | -6.8 |
|  | Harari | 13,435.1 (9,679.1, 16,559.1) | 1,323.5 (8,89.7, 1,945.4) | -7.5 |
|  | Oromia | 123,62.1 (9,604.0, 14,884.5) | 1,595.3 (1,326.5, 1,935.4) | -6.6 |
|  | Somali | 9,934.2 (7,110.2, 15,086.9) | 3,527.9 (2,680.0, 4,638.5) | -3.3 |
|  | Southern Nations, Nationalities, and Peoples | 12,503.5 (10,047.1, 14,871.9) | 2,065.8 (1,641.9, 2,558.5) | -5.8 |
|  | Tigray | 12,559.9 (9,638.4, 15,252.4) | 1,579.7 (1,231.0, 1,982.5) | -6.7 |
| Kenya | Baringo | 7560.0 (4206.7, 11168.9) | 3679.1 (2187.4, 5228.3) | -2.3 |
|  | Bomet | 1674.4 (822.6, 3565.2) | 2035.0 (881.4, 3277.7) | 0.6 |
|  | Bungoma | 3940.6 (2186.0, 6231.4) | 1477.6 (947.9, 2039.6) | -3.2 |
|  | Busia | 4591.0 (2375.0, 8025.4) | 2538.5 (1487.9, 3909.3) | -1.9 |
|  | Elgeyo-Marakwet | 5228.8 (2871.9, 8190.2) | 2239.3 (1418.6, 3231.4) | -2.7 |
|  | Embu | 2675.7 (1477.1, 4826.9) | 1952.9 (1094.2, 3089.3) | -1.0 |
|  | Garissa | 7886.5 (4430.4, 13280.4) | 6023.9 (3456.7, 9824.4) | -0.9 |
|  | Homa Bay | 6226.4 (3486.7, 9646.5) | 3016.4 (1574.8, 4543.8) | -2.3 |
|  | Isiolo | 9141.1 (5402.0, 13401.4) | 4108.4 (2378.5, 6154.9) | -2.6 |
|  | Kajiado | 3798.5 (2031.9, 6722.2) | 2185.2 (1188.9, 3333.2) | -1.8 |
|  | Kakamega | 5199.6 (2951.3, 7796.4) | 3173.4 (1845.0, 4588.0) | -1.6 |
|  | Kericho | 2844.6 (1369.5, 5513.9) | 1347.7 (834.2, 2097.4) | -2.4 |
|  | Kiambu | 4755.0 (2684.9, 6857.8) | 2842.7 (1493.9, 3903.4) | -1.7 |
|  | Kilifi | 4129.3 (2334.7, 7157.8) | 2448.7 (1528.8, 3556.4) | -1.7 |
|  | Kirinyaga | 3086.8 (1683.6, 5479.5) | 2519.7 (1348.1, 3745.9) | -0.7 |
|  | Kisii | 6424.3 (3822.1, 8887.5) | 3220.5 (1998.0, 4334.9) | -2.2 |
|  | Kisumu | 6143.6 (3054.3, 9887.9) | 3705.9 (1640.8, 5538.2) | -1.6 |
|  | Kitui | 3850.8 (2195.3, 6393.9) | 2444.7 (1295.1, 3925.4) | -1.5 |
|  | Kwale | 4872.4 (2820.3, 8263.1) | 3234.4 (1889.2, 4823.2) | -1.3 |
|  | Laikipia | 2673.1 (1460.0, 4963.8) | 1515.3 (807.2, 2383.4) | -1.6 |
|  | Lamu | 4746.8 (2701.3, 7953.3) | 3185.2 (1902.1, 4997.1) | -1.3 |
|  | Machakos | 2262.8 (1209.6, 4097.9) | 1760.9 (1067.6, 2614.7) | -0.8 |
|  | Makueni | 2847.0 (1626.0, 4877.9) | 1863.6 (918.9, 3068.5) | -1.4 |
|  | Mandera | 7864.1 (3970.6, 13894.9) | 12190.3 (5751.5, 18338.0) | 1.4 |
|  | Marsabit | 4931.2 (2533.1, 8747.2) | 4100.4 (2084.8, 6788.0) | -0.6 |
|  | Meru | 2838.1 (1526.9, 5163.7) | 3030.3 (1806.9, 4520.2) | 0.2 |
|  | Migori | 6304.8 (3617.1, 9774.8) | 3723.1 (2105.1, 5576.4) | -1.7 |
|  | Mombasa | 3353.3 (1784.7, 5858.3) | 2124.3 (1173.3, 3147.2) | -1.5 |
|  | Murang'a | 2761.3 (1457.8, 4836.6) | 2238.6 (1135.4, 3277.7) | -0.7 |
|  | Nairobi | 3719.5 (1995.1, 5294.4) | 2250.1 (1082.8, 3132.0) | -1.6 |
|  | Nakuru | 3631.0 (1816.5, 6511.0) | 1748.3 (991.9, 2601.5) | -2.4 |
|  | Nandi | 5001.2 (2543.9, 8654.5) | 2747.5 (1372.9, 4090.3) | -1.9 |
|  | Narok | 5830.6 (2977.5, 10028.2) | 4886.7 (2632.5, 7273.5) | -0.6 |
|  | Nyamira | 5047.1 (2816.0, 7492.9) | 4705.2 (2768.1, 6556.4) | -0.2 |
|  | Nyandarua | 4918.3 (2887.7, 7448.0) | 4763.2 (2762.9, 6770.8) | -0.1 |
|  | Nyeri | 3318.4 (1939.7, 5128.0) | 2500.1 (1561.3, 3475.1) | -0.9 |
|  | Samburu | 4401.2 (2143.0, 7819.0) | 3999.2 (1973.7, 6936.8) | -0.3 |
|  | Siaya | 5986.0 (3476.2, 9176.5) | 1789.2 (1031.9, 2796.5) | -3.9 |
|  | Taita Taveta | 3508.2 (1910.7, 6459.2) | 3085.6 (1838.3, 4524.7) | -0.4 |
|  | Tana River | 8229.4 (4803.0, 14072.9) | 6928.6 (4246.3, 9860.1) | -0.6 |
|  | Tharaka Nithi | 6193.8 (3664.2, 9681.4) | 1821.4 (1082.9, 2863.9) | -4.0 |
|  | Trans Nzoia | 2776.4 (1403.9, 5304.9) | 1906.6 (983.1, 2968.1) | -1.2 |
|  | Turkana | 5796.2 (2786.4, 10293.3) | 4946.8 (2350.5, 9095.2) | -0.5 |
|  | Uasin Gishu | 2536.1 (1369.5, 4916.6) | 1544.5 (917.7, 2284.7) | -1.6 |
|  | Vihiga | 4038.1 (2484.8, 6170.8) | 3129.9 (2016.4, 4498.5) | -0.8 |
|  | Wajir | 8415.9 (4092.5, 13999.3) | 7667.3 (4025.1, 12227.5) | -0.3 |
|  | West Pokot | 5516.9 (3104.5, 9266.7) | 3641.8 (2207.8, 5741.3) | -1.3 |
